# Development of [^18^F]FAMTO: A novel fluorine-18 labelled positron emission tomography (PET) radiotracer for imaging CYP11B1 and CYP11B2 enzymes in adrenal glands

**DOI:** 10.1016/j.nucmedbio.2018.11.002

**Published:** 2019

**Authors:** Salvatore Bongarzone, Filippo Basagni, Teresa Sementa, Nisha Singh, Caleb Gakpetor, Vincent Faugeras, Jayanta Bordoloi, Antony D. Gee

**Affiliations:** a School of Imaging Sciences & Biomedical Engineering, 4th floor Lambeth Wing, St Thomas' Hospital, King's College London, London SE1 7EH, United Kingdom; b Department of Neuroimaging, Institute of Psychiatry, King's College London, London SE5 8AF, United Kingdom

**Keywords:** Metomidate, Adrenal glands, Fluorine-18 radiochemistry, Primary aldosteronism, CYP11B2, Positron emission tomography

## Abstract

**Introduction:**

Primary aldosteronism accounts for 6–15% of hypertension cases, the single biggest contributor to global morbidity and mortality. Whilst ~50% of these patients have unilateral aldosterone-producing adenomas, only a minority of these have curative surgery as the current diagnosis of unilateral disease is poor. Carbon-11 radiolabelled metomidate ([^11^C]MTO) is a positron emission tomography (PET) radiotracer able to selectively identify CYP11B1/2 expressing adrenocortical lesions of the adrenal gland. However, the use of [^11^C]MTO is limited to PET centres equipped with on-site cyclotrons due to its short half-life of 20.4 min. Radiolabelling a fluorometomidate derivative with fluorine-18 (radioactive half life 109.8 min) in the *para*-aromatic position ([^18^F]FAMTO) has the potential to overcome this disadvantage and allow it to be transported to non-cyclotron-based imaging centres.

**Methods:**

Two strategies for the one-step radio-synthesis of [^18^F]FAMTO were developed. [^18^F]FAMTO was obtained *via* radiofluorination *via* use of sulfonium salt (**1**) and boronic ester (**2**) precursors. [^18^F]FAMTO was evaluated *in vitro* by autoradiography of pig adrenal tissues and *in vivo* by determining its biodistribution in rodents. Rat plasma and urine were analysed to determine [^18^F]FAMTO metabolites.

**Results:**

[^18^F]FAMTO is obtained from sulfonium salt (**1**) and boronic ester (**2**) precursors in 7% and 32% non-isolated radiochemical yield (RCY), respectively. Formulated [^18^F]FAMTO was obtained with >99% radiochemical and enantiomeric purity with a synthesis time of 140 min from the trapping of [^18^F]fluoride ion on an anion-exchange resin (QMA cartridge). *In vitro* autoradiography of [^18^F]FAMTO demonstrated exquisite specific binding in CYP11B-rich pig adrenal glands. *In vivo* [^18^F]FAMTO rapidly accumulates in adrenal glands. Liver uptake was about 34% of that in the adrenals and all other organs were <12% of the adrenal uptake at 60 min post-injection. Metabolite analysis showed 13% unchanged [^18^F]FAMTO in blood at 10 min post-administration and rapid urinary excretion. *In vitro* assays in human blood showed a free fraction of 37.5%.

**Conclusions:**

[^18^F]FAMTO, a new ^18^F-labelled analogue of metomidate, was successfully synthesised. *In vitro* and *in vivo* characterization demonstrated high selectivity towards aldosterone-producing enzymes (CYP11B1 and CYP11B2), supporting the potential of this radiotracer for human investigation.

## 1 Introduction

The most common secondary cause of hypertension is primary aldosteronism (PA), which is reported in approximately 6–15% of all hypertensive patients. By the year 2025, PA is expected to directly impact one in four people with hypertension globally [[1], [2], [3], [4], [5], [6], [7]]. PA is characterized by an excess secretion of aldosterone, steroid hormone with mineralocorticoid activity produced by the zona glomerulosa of the adrenal cortex where aldosterone synthase (CYP11B2) enzymes play an essential role in aldosterone production [3,8,9]. Aldosterone increases sodium reabsorption and potassium excretion in the renal distal tubules and collecting ducts of nephrons, influencing water retention and blood pressure [3].

Therefore, pathological conditions showing an overproduction of aldosterone frequently lead to hypertension and hypokalaemia resulting in detrimental effects to an individual's cardiovascular system [1]. Over 90% of all PA patients have a sporadic form [10]. The two most common subtypes of sporadic PA are unilateral aldosterone-producing adenomas (UAPA, 40%) or bilateral adrenal hyperplasia (BAH, 60%) [5,11]. Correctly differentiating between the unique features of UAPA and BAH is crucial in identifying an appropriate intervention. UAPA is curable through surgical removal of the diseased adrenal whilst BAH is treated pharmacologically with mineralocorticoid receptor antagonists (*e.g.* spironolactone) [12]. The most significant obstacle for decision makers is the need to distinguish UAPA from other causes of PA such as BAH or non-functioning adrenal adenoma (incidentalomas) [13]. Invasive bilateral adrenal vein sampling (AVS) is the current gold-standard diagnostic procedure adopted, with cannulation success rates varying from 8% to 95% [10,[14], [15], [16]].

Computed tomography (CT) and magnetic resonance imaging (MRI) have been used for non-invasive lateralisation of aldosterone hypersecretion. These show low diagnostic accuracy due to suboptimal spatial resolution and sensitivity to detect small UAPA (<1 cm) [14,[17], [18], [19], [20]]. Positron emission tomography (PET) is considered an accurate and non-invasive alternative to AVS in the management of patients with PA and adrenal adenoma [21].

**Fig. 1:**
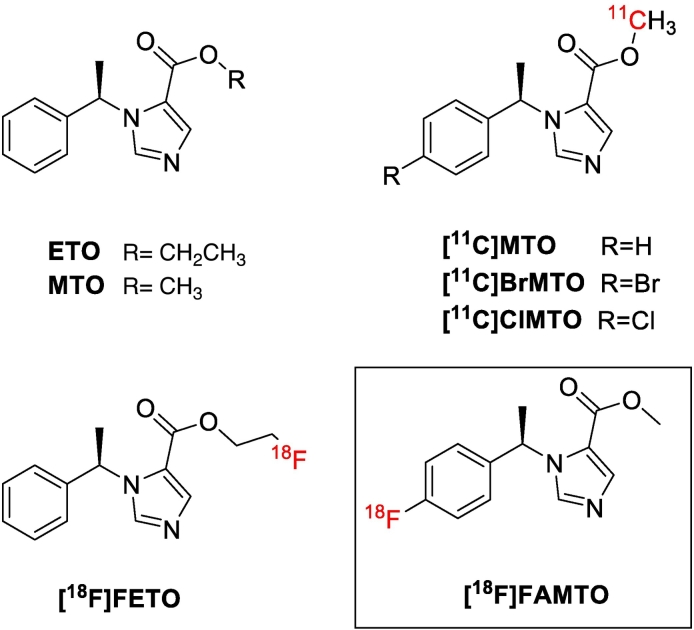
Chemical structures of ETO, MTO, [^11^C]MTO, [^11^C]BrMTO, [^11^C]ClMTO, [^18^F]FETO, and [^18^F]FAMTO.

Methyl and ethyl esters of 1-[(1*R*)-1-phenylethyl]-1H-imidazole-5-carboxylic acid, such as (*R*)-metomidate (MTO, Fig. 1) and (*R*)-etomidate (ETO), are a class of compounds that interact with the mitochondrial cytochrome P-450 enzymes (11β-hydroxylase (CYP11B1) and CYP11B2) in the adrenal cortex (IC_50_ for CYP11B1 = 0.24 ± 0.05 nM (MTO) and 0.5 ± 0.2 nM (ETO), IC_50_ for CYP11B2 = 0.59 ± 0.64 nM (MTO) and 1.7 ± 0.9 nM (ETO)) [[22], [23], [24]]. MTO and ETO have been used as template to develop PET radiolabelled radiotracers using β^+^-emitting radionuclides such as ^11^C and ^18^F [21,25,26].

Bergström et al. have developed [^11^C]MTO (Fig. 1) by radiolabelling the methyl ester group with ^11^C. [^11^C]MTO possesses high specific binding to adrenal enzymes *in vitro* and in non-human primate studies [25,27,28]. Blocking experiments using ETO (10 μM) confirmed the specific uptake of [^11^C]MTO to pig adrenal sections [25]. In humans [^11^C]MTO showed high uptake in adrenal glands (SUV ratio of adrenal gland/liver ratio 1.5 ± 0.7, 15–45 min) and high standardised uptake values (SUV) in aldosterone-hypersecreting adenomas [25,27,[29], [30], [31]].

Metabolite studies of [^11^C]MTO in humans revealed that unchanged [^11^C]MTO accounted for 40% and 28% of total blood-borne radioactivity at 20 min and 40 min post-injection, with two polar metabolites present in the plasma [30]. Although [^11^C]MTO has good imaging properties for visualisation of UAPA [21], novel radiotracers with increased adrenal-to-liver ratio (large liver uptake hampers the assessment of the right adrenal) and a longer half life radionuclide (*e.g.* ^18^F) are needed for a more widespread adoption of this diagnostic approach.

An increased adrenal-to-liver PET signal was achieved by adding a halogen atom (*e.g.* chlorine or bromine) in the para-position of MTO's benzene group such as chlorine ([^11^C]ClMTO, Fig. 1) and bromine ([^11^C]BrMTO, Fig. 1). In rats [^11^C]MTO gives an adrenal-to-liver ratio of 3.8 at 30 min post-injection, increasing to 4.4 and 7.2 using [^11^C]ClMTO and [^11^C]BrMTO, respectively [32]. [^11^C]MTO is not used in a widespread manner, partly because of its short half-life that limits its use to PET centres with on-site cyclotrons. A MTO analogue with a longer fluorine-18 half-life would enable the examination of more patients per tracer production, and its distribution to PET-centres without on-site cyclotrons.

Wadsak and Mitterhauser developed an ETO derivative radiolabelled with fluorine-18 ([^18^F]FETO, Fig. 1) to enhance the image resolution, and pharmacokinetic and pharmacodynamic properties of [^11^C]MTO [33]. MTO and FETO have similar binding affinities to displace the radioligand 4-[^131^I]iodometomidate ([^131^I]IMTO) on homogenized rat adrenal membranes as an acceptable surrogate for testing inhibitory affinity *versus* CYP11B1 and CYP11B2 (IC_50_ = 3.69 ± 1.92 nM and 2.9 ± 0.55 nM, respectively) [34]. [^18^F]FETO was produced using an automated one-step protocol within 70 min (total synthesis time) with a non-isolated radiochemical yield (RCY) of 20 ± 3% [35]. Regional organ distribution of [^18^F]FETO in rat revealed an adrenal-to-liver ratio of 14.48 at 60 min post-injection [36]. Metabolite analysis indicated that [^18^F]FETO is degraded to 2-[^18^F]fluoroethanol *in vitro* and *in vivo* [37]. In humans, [^18^F]FETO is rapidly metabolised in the first 10 min (91% intact [^18^F]FETO after 2 min; 24% after 10 min, 11% after 20 min and 3.7% after 90 min) [37]. [^11^C]MTO and [^18^F]FETO showed rapid metabolism *in vivo* corresponding to de-esterification to the non-radioactive and inactive carboxylic acid derivative (IC_50_ = 0.12 mM) [34,38] and radioactive metabolites, such as [^11^C]methanol and [^11^C]formaldehyde for [^11^C]MTO and [^18^F]fluoroethanol for [^18^F]FETO, affecting the signal/noise ratio and specificity of the imaging data acquired [30,36,37].

A *para*-fluorinated aromatic (*R*)-MTO derivative (FAMTO, Fig. 1) has comparable affinity for adrenal enzymes (K_i_ = 7.3 nM measured using [^131^I]IMTO) as MTO (K_i_ = 4.02 ± 1.87 nM) in rat adrenal membranes [39]. We therefore developed a radiosynthetic strategy to produce a ^18^F-radiolabelled analogue of MTO ([^18^F]FAMTO) in order to evaluate it as a potential radiotracer for aldosterone-producing adenomas.

## 2 Materials & methods

### 2.1 General consideration

[^18^F]Fluoride ion was produced using an RDS112 cyclotron at King's College London PET Centre by the ^18^O(p,n)^18^F reaction *via* proton irradiation of enriched (95%) ^18^O water.

### 2.2 Trapping and releasing of [^18^F]fluoride ion on a Sep-Pak Accell Plus QMA cartridge

0.5–1.5 GBq of aqueous [^18^F]fluoride ion was trapped in an anion-exchange resin cartridge (Sep-Pak Accell Plus QMA cartridge, WAT023525, Waters) pre-activated with 10 mL 1 N NaHCO_3_ (aq), 10 mL water (H_2_O) followed by 10 mL air. The trapped [^18^F]fluoride ion was released with solution A, composed of 25.5 μmol Kryptofix (K_222_) and 4.5 μmol KHCO_3_ in acetonitrile (MeCN):H_2_O (85:15, 1 mL) or solution B, composed of 19.55 μmol K_222_ and 0.9 μmol K_2_CO_3_ in MeCN:H_2_O (85:15, 1 mL). The solvent was removed by heating at 90 °C under a stream of N_2_ and fluoride ion was dried by azeotropic distillation with MeCN (2 × 0.5 mL). Compound **1** (5 mg) was dissolved in anhydrous dimethyl sulfoxide (DMSO, 100–500 μL) and the obtained solution was added to the dried [^18^F]fluoride ion/K_222_/K_2_CO_3_ mixture. The vial was then sealed and stirred at 110–180 °C for 15–30 min, quenched with H_2_O and analysed by radioTLC.

### 2.3 Radioynthesis of [^18^F]FAMTO

#### 2.3.1 High base procedure

The trapped [^18^F]fluoride ion was released with 0.5 mL of a solution composed of K_222_ (16 μmol) and K_2_CO_3_ (2.4 μmol) in MeCN:H_2_O (80:20). Azeotropic drying was performed by addition of MeCN (2 × 0.5 mL) and heating at 90 °C under a stream of N_2_. To the dried mixture, 500 μL of DMF was added. Aliquots of 500, 250, 125 and 87 μL of [^18^F]KF/K_222_/K_2_CO_3_, **2** (6.3 mg, 18 μmol) and tetrakis(pyridine)copper(II) triflate (Cu(OTf)_2_(py)_4_, 3.6 mg, 5 μmol) were added in a vial. DMF was added to achieve a final volume of 500 μL (see Table 2). Radiofluorination was conducted at 118 °C for 20 min, cooled to room temperature, and diluted with H_2_O.

#### 2.3.2 Low base procedure

Aqueous [^18^F]fluoride ion (100–1000 MBq) was loaded onto a QMA cartridge, the cartridge was flushed with air and the ^18^F elution was performed using 2 mL of K_222_ (1.5 μmol) and K_2_CO_3_ (0.87 μmol) in MeCN:H_2_O (80:20). Azeotropic drying was performed by addition of MeCN (2 × 1 mL) and heating at 90 °C under a stream of N_2_. To the dried mixture DMF (500, 300, 200, and 100 μL), **2** (6.3 mg, 18 μmol) and Cu(OTf)_2_(py)_4_ (3.6 mg, 5 μmol) were added (see Table 2). Radiofluorination was conducted at 118 °C for 20 min under air, cooled to room temperature, and diluted with H_2_O.

### 2.4 Purification and analysis of [^18^F]FAMTO

The crude mixture was purified by semi-preparative HPLC (see Appendix A). The purified [^18^F]FAMTO was subsequently diluted with 40 mL of PBS, the solution loaded onto a Sep-Pak tC18 Plus Long SPE Cartridge 900 mg, 37–55 μm (cat. no. WAT036800, Waters) and eluted with ethanol (2 mL). Ten fractions (0.2 mL) were collected and radioactivity determined. Fractions with highest radioactivity were combined and formulated with saline. The formulated solution (4–6 mL of 10% ethanol in saline) containing 1–10 MBq of [^18^F]FAMTO was used for *in vitro* and *in vivo* experiments. Analytical reverse-phase HPLC (see Appendix A) was used to determine the molar activity, radiochemical and chemical purity. Identification of the radioactive products was confirmed by co-elution of added non-radioactive compounds.

### 2.5 Determination of chiral conformation and enantiomeric excess of 2,4 and [^18^F]FAMTO

Circular dichroism (CD) spectra of (*R*)-ETO (enantiomeric excess (e.e.) >99%, Sigma Aldrich), (*R*)-**2**, (*R*)-**4** and (*S*)-**8** (e.e. >97%, Sigma Aldrich) were acquired on the Applied Photophysics Ltd. Chirascan Plus spectrometer (Fig. S1).

Enantiomeric purity of (*R*)-**2**, (*R*)-**4**, (*R*)-**5**, and (*R*)-[^18^F]FAMTO was performed on a chiral HPLC Lux 5 μm Cellulose-3250 × 4.6 mm coloumn (Table S2). Commercial (*R*)-ETO (e.e. >99%) and (*S*)-**8** (e.e. >97%) were used as control.

### 2.6 Determination of RCY and molar activity of [^18^F]FAMTO

The entire quenched reaction mixture was used to determine non-isolated and isolated RCY.

All RCYs are reported as decay corrected values [40]. Non-isolated RCYs were determined by integrating the area under the curve in the preparative radio-HPLC chromatogram or iTLC. Isolated RCY were calculated by relating the amount of isolated [^18^F]FAMTO to the amount of [^18^F]fluoride ion trapped on QMA cartridge.

The molar activities (GBq/μmol) were calculated by dividing the radioactivity of [^18^F]FAMTO by the amount of the unlabeled FAMTO determined from the peak area in the UV-HPLC chromatograms (λ = 254 nm, common wavelength for identifying metomidate derivative compounds such as etomidate and iodometomidate [25,38], Fig. S1). FAMTO concentrations were determined from a UV-absorbance calibration curve (see Appendix A).

### 2.7 Pig organ tissues

Pig tissues were purchased from Seralab, UK. Adrenal glands, kidneys, and liver were collected from an healthy animal (female, 9 months old). Tissues were delivered at 4 °C in Dulbecco's media. Once collected, tissues were cut in small and medium pieces. Pieces were snap frozen in an isopentane bath cooled to −30 °C and stored at −80 °C.

### 2.8 Autoradiography

**Fig. 2:**
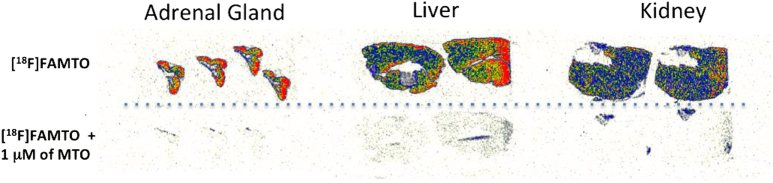
Frozen tissue-section autoradiography of [^18^F]FAMTO (1 nM) binding to pig adrenal, pig liver, pig kidney. Lower panel [^18^F]FAMTO (1 nM) blocking studies using 1 μM MTO.

Frozen sections (20 μm) of pig adrenal glands, kidneys, and liver were prepared in a Cryostat and put on superfrost glass slides. At the start of the experiment, the slides warmed to room temperature and preincubated for 10 min in TRIS buffer (50 mM, pH 7.4). Slides were then transferred to containers containing 1 nM of [^18^F]FAMTO in 40 mL of TRIS buffer (50 mM, pH 7.4). In a duplicate set of containers, 1 μM of MTO was added to block specific binding. After incubation for 30 min (the slides were washed 2 × 3 min in fresh 50 mM TRIS buffer, pH 7.4). The slides were dried and then exposed to phosphor imaging plates (BAS-IP TR 2040, GE Healthcare, UK) for 30 min and scanned in a Typhoon 8600 phosphorimager (GE Healthcare, UK). Images (Fig. 2) were analysed using OptiQuant 5.0 software (PerkinElmer, UK).

### 2.9 Animal studies

Biodistribution and metabolite studies were carried out in adult male Sprague-Dawley rats (300–400 g). All animal studies were carried out in accordance with the UK Home Office Animals (Scientific Procedures) Act 1986. Experiments complied with UK Research Councils' and Medical Research Charities' guidelines on responsibility in the use of animals in bioscience research, under UK Home Office project and personal licenses.

### 2.10 *Ex vivo* biodistribution

**Fig. 3:**
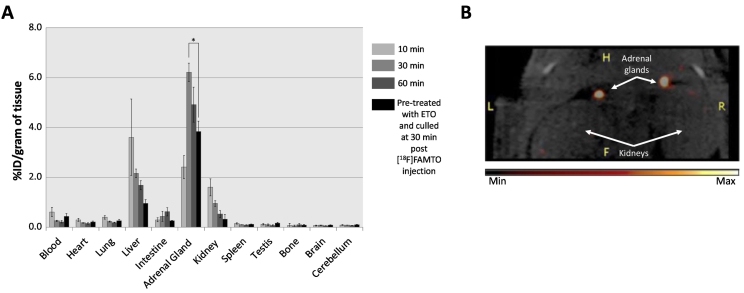
(A) Organ uptake of [^18^F]FAMTO (i.v.) in male Sprague Dawley rats (*n* = 3, for each group) at 10, 30, 60 min post-injection. Rats pre-treated with ETO (1 mg/kg, i.v.) 15 min prior to [^18^F]FAMTO i.v. injection and culled after 30 min. A two-tailed paired Student's *t*-test was used to compare adrenal-liver uptake and adrenal uptake in rats pre-treated with ETO *versus* that of control rats. (B) *In vivo* PET imaging of a male Sprague Dawley rats showing adrenal uptake of [^18^F]FAMTO after pre-treatment with ETO (1 mg/kg).

Nine anaesthetised rats (2–3% isoflurane/oxygen) were injected intravenously with 1–3 MBq/kg of formulated [^18^F]FAMTO and sacrificed at 15, 30 and 60 min post-injection. The specific uptake of [^18^F]FAMTO was evaluated in three anaesthetised rats (2–3% isoflurane/oxygen) using 1 mg/kg ETO i.v. (95% saline and 5% ethanol) 15 min before [^18^F]FAMTO injection (1–3 MBq/kg), and animals sacrificed 30 min post-radiotracer injection. Tissues including whole brain, heart, lungs, stomach, liver, spleen, small intestine, large intestine, kidneys, bladder, thigh bone, adrenal glands, and testes were excised. Urine and blood were also collected. All samples were weighed and radioactivity content measured using an automated well counter with a standard dilution of [^18^F]FAMTO. Counts were decay-corrected and the %ID/g calculated (Fig. 3A and Table S1). Data are expressed as mean ± standard error (SE) from three independent replicates, unless otherwise indicated. Statistical analysis of %ID/g in the biodistribution study was performed with IBM SPSS Statistics (version 24). Student *t*-tests were used to determine statistical significance with *p* < 0.05 considered significant.

### 2.11 Analysis of radiolabelled metabolites in rats

**Fig. 4:**
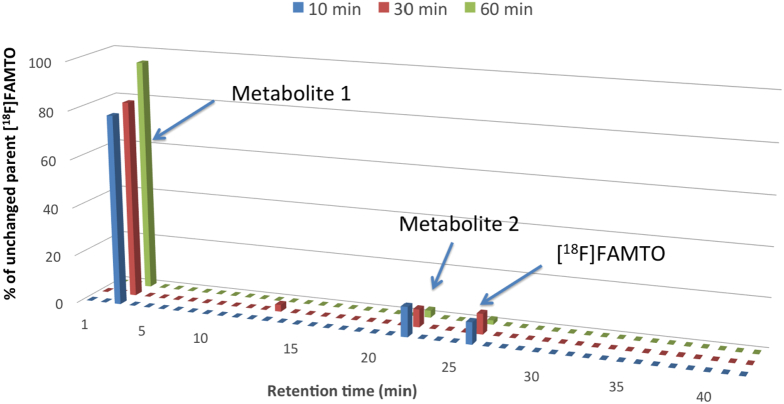
Plasma metabolite analysis of [^18^F]FAMTO in male Sprague Dawley rats at 10, 30, 60 min post-radiotracer injection.

**Scheme 1:**
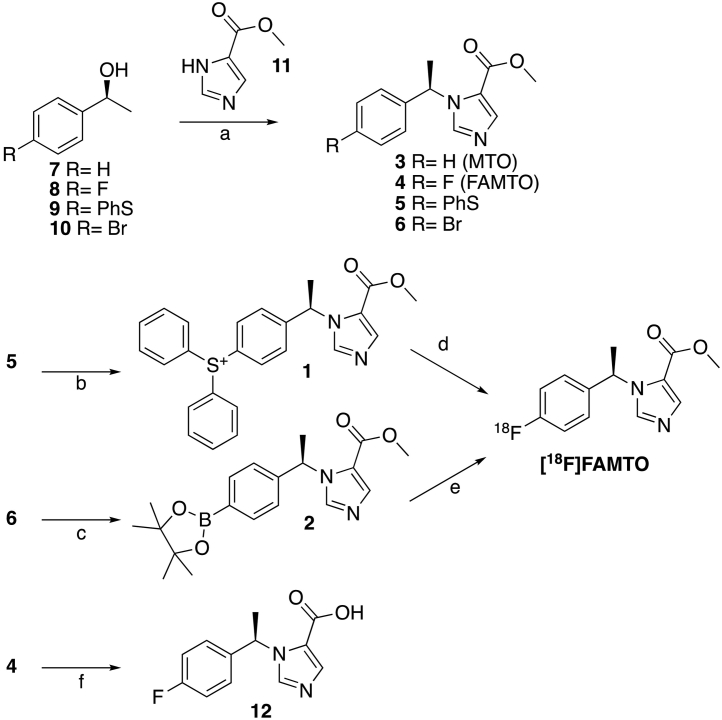
Reaction scheme for production of MTO, FAMTO, **12**, and [^18^F]FAMTO. a: DtBAD, PPh_3_, THF, 0 °C, rt., 17 h; b: Ph_2_ITfO, Cu(C_6_H_5_COO)_2_ TFSA, PhCl, 125 °C, 2 h; c: (BPin)_2_, KOAc, (C_17_H_14_P)_2_Fe·PdCl_2_, DMSO, 80 °C, 15 h; d: [^18^F]fluoride ion, DMSO, 110 °C, 14–30 min; e: [^18^F]fluoride ion, Cu(Py)_4_(OTf)_2_, DMF, 110 °C, 20 min; f: NaOH 2 N, MeOH, rt., 17 h.

Blood and urine samples were collected at 10, 30, and 60 min post-injection to measure the amounts of unchanged tracer and radioactive metabolites. Blood was centrifuged at 6000 rpm for 5 min, the supernatant was treated with MeCN (1:1) and centrifuged to precipitate plasma proteins. Plasma supernatant and urine samples were then injected into an HPLC semipreparative column (see Appendix A), and the eluate collected in 1.5 mL test tubes and gamma-counted. Two metabolites (t_R_ = 3 and 21 min) and [^18^F]FAMTO were observed in the plasma (Fig. 4). One metabolite (t_R_ = 3 min) was observed in the urine (Fig. S2). 2.5 μL aliquots of radioactivity eluting at t_R_ = 3 min and compound **12** (Scheme 1) were analysed by TLC plates (Merck F-254 silica gel) eluted with dichloromethane:methanol:trifluoroacetic acid (TFA) (8:2:0.05). TLC plates were scanned in a Typhoon 8600 phosphorimager (GE Healthcare, UK) and analysed using OptiQuant 5.0 software (PerkinElmer, UK).

### 2.12 Stability studies in plasma

Human blood (1 mL) was incubated with 0.7–1 MBq (200 μL) [^18^F]FAMTO at room temperature for 10 and 20 min. After incubation, plasma was separated by centrifugation (5 min, 4000 ×*g*) and the proteins were precipitated by the addition of an equal volume of MeCN. The tubes were centrifuged for 5 min at 4000 ×*g*. The supernatant was then injected onto the HPLC (HPLC method 1) and serial 1.5 mL HPLC fractions were collected and gamma-counted. [^18^F]FAMTO (0.7–1 MBq, 200 μL) incubated in PBS (1 mL) was used as control.

### 2.13 Plasma protein binding

The plasma protein binding of [^18^F]FAMTO was measured in the plasma of blood samples. Plasma was prepared from fresh human blood by centrifugation (5 min, 4000 ×*g*) at room temperature. Two vials containing fresh human plasma (300 μL) and PBS (300 μL, control) were incubated at room temperature with 30 μL of a solution containing [^18^F]FAMTO (1–2 MBq). After 10 min, each solution was sampled and counted. The solution (200 μL) was injected into a filter device (Millipore Centrifree tubes, YM-30) and subsequently centrifuged for 5 min at 6000 rpm. The solution passed through a centrifugal filters unit (nominal molecular weight limit 30,000 Da) and the filtrate and control samples were gamma-counted.

### 2.14 PET imaging

PET-CT scans were performed on a BioScan nanoPET-CT® PLUS (Mediso, Hungary) scanner using their proprietary acquisition software (Nucline 1.07). The rats were anaesthetised and maintained at 2.5% isofluorane and 1 L/min flow of Oxygen (O_2_). A CT scan was acquired prior to the commencement of the PET scan. Dynamic PET scans were acquired for 60 min post-intra-venous (IV) administration by tail vein injection of [^18^F]FAMTO (1.0 MBq, *n* = 2) or by pre-administration ETO (1 mg/kg) 15 min prior to [^18^F]FAMTO injection (1.0 MBq, *n* = 1).

PET scans were reconstructed using Tera-Tomo 3D image reconstruction algorithm. Reconstructions were performed with the detector coincidence mode set at 1:3. Corrections for decay, randoms, crystal dead time, detector normalization and, attenuation correction was implemented. A total of 6 subsets and 4 iterations were applied resulting in a voxel size of 0.25 × 0.25 × 0.25 mm^3^ for CT, and 0.4 × 0.4 × 0.4 mm^3^ for PET. The PET and CT images were co-registered automatically. Image analysis was performed using pre-clinical image postprocessing software VivoQuant™.

## 3 Results

### 3.1 Chemistry

Aryl sulfonium salt **1** (Scheme 1) was synthesised following the procedure reported by Sander et al. [41]. The thioether **5** reacted with diaryliodonium triflate under copper(II) catalysis (125 °C, 1 h) giving **1** in 53% yield. Pinacol boronate ester **2** was prepared by the following procedure adapted from the literature [42]. The *para*-bromo MTO derivative (**6**) reacted with bis(pinacolato)diboron in the presence of potassium acetate and Pd-catalyst to form **2** with a yield of 76 ± 10% (*n* = 3).

Scheme 1 shows the route used to synthesise **3**–**6** *via* Mitsunobu reaction that involves the use of both an oxidising agent such as di-tert-butyl azodicarboxylate (DtBad) and a reducing agent such as triphenylphosphine (PPh_3_) under mild conditions. Yields between 40 and 60% were obtained.

### 3.2 Radiosynthesis of [^18^F]FAMTO

Two ^18^F-labeling strategies were selected to produce [^18^F]FAMTO, the first *via* an aryl sulfonium salt (**1**, Scheme 1) and the second *via* an aryl boronic precursor (**2**).

The strategy of labeling sulfonium salts with ^18^F fluoride ion has been reported to be a direct, straightforward nucleophilic substitution [41]. Following the optimized procedure developed by Sander et al., dimethyl sulfoxide (DMSO), potassium bicarbonate (KHCO_3_, 30 mM), Kryptofix (K_222_, 30 mM) and **1** (5 mg) in 500 μL DMSO at 110 °C for 15 min, [^18^F]FAMTO was obtained with very low non-isolated radiochemical yield (RCY, 1%, entry 1, Table 1).

**Table 1:** Radiosynthesis of [^18^F]FAMTO from **1**.

Entry	Releasing solution	Time (min)	Volume (mL)	Temperature (°C)	Non-isolated RCY (%)^c^
1	A^a^	15	500	110	1 ± 0.5 (n = 3)
2	A	15	250	110	0 (n = 2)
3	A	30	250	110	0 (n = 2)
4	B^b^	30	500	110	8 (n = 1)
5	B	30	250	110	7 ± 2 (n = 3)
6	B	30	100	110	1, 4 (n = 2)
7	B	30	250	150	0 (n = 1)
8	B	30	250	180	0 (n = 1)

a Solution A: 0.5 mL K_222_ (30 mM) and KHCO_3_ (30 mM) in MeCN:H_2_O (85:15).

b Solution B: 1 mL of K_222_ (23 mM) and K_2_CO_3_ (6 mM) in MeCN:H_2_O (85:15).

c Determined by radio-TLC (% ± SEM).

Screening of solvent volume, time and bases commonly used for nucleophilic substitution reactions with [^18^F]fluoride ion was investigated in an attempt to improve the RCY. Increasing the concentration of precursor from 0.01 mg/mL to 0.02 mg/mL (entry 2) or increasing the reaction time from 15 to 30 min (entry 3) did not give the desired product. By substituting KHCO_3_ with K_2_CO_3_, [^18^F]FAMTO was obtained using 500 and 250 μL of DMSO with a non-isolated RCY of 8% and 7%, respectively (entries 4–5). Decreasing further the volume of DMSO to 100 μL caused a RCY drop (entry 6). Increasing the temperature from 110 to 150 or 180 °C, afforded no [^18^F]FAMTO whatsoever.

An alternative radiolabelling strategy to produce [^18^F]FAMTO was subsequently investigated starting from the aryl boronic derivative **2**. Initially, the experiment was performed by mixing a solution of **2** and Cu(OTf)_2_(py)_4_ in DMF with the dried residue of [^18^F]fluoride ion/K_222_/K_2_CO_3_ and heated at 110 °C for 20 min under air. The formation of [^18^F]FAMTO under these conditions was not observed (entry 1, Table 2).

**Table 2:** Optimization of radiofluorination of **2** to form [^18^F]FAMTO.

Entry^a^	Releasing solution		Activity released from QMA (%)	Reaction			Non-isolated RCY (%)^b^	Isolated RCY (%)^c^
	K_222_ (μmol)	K_2_CO_3_ (μmol)		K_222_ (μmol)	K_2_CO_3_ (μmol)	Volume (DMF)		
1	20	12	95 ± 4 (*n* = 8)	20	12	500	0 (n = 2)	n.d.
2	20	12		10	6	500	17, 16 (n = 2)	n.d.
3	20	12		5	3	500	18, 16 (n = 2)	n.d.
4	20	12		3	1.5	500	22, 23 (n = 2)	n.d.
5	1.5	0.87	82 ± 2 (*n* = 27)	1.5	0.87	500	32 ± 6 (n = 5)	28 ± 14 (*n* = 5)
6	1.5	0.87		1.5	0.87	300	29 ± 7 (*n* = 3)	n.d.
7	1.5	0.87		0.87	200	200	32 ± 2 (n = 18)	18 ± 2 (*n* = 18)
8	1.5	0.87		0.87	150	150	11 (n = 1)	n.d.

a **2** (18 μmol), Cu(OTf)_2_(py)_4_ (5 μmol), DMF (150–500 μL), 20 min, 110 °C.

b Estimated by analytical radio-HPLC of the crude product (% ± SEM).

c Calculated from the amount of isolated [^18^F]FAMTO to the initial amount of [^18^F]fluoride ion trapped in the Sep-Pak Accell Plus QMA cartridge (% ± SEM).

Neumaier and coworkers have reported an efficient protocol to increase the RCY of copper-mediated aromatic radiofluorination using a “low amount of base” strategy [43]. Indeed using small amount of K_222_/K_2_CO_3_ in DMF obtained by aliquoting the dried residue of [^18^F]fluoride ion/K_222_/K_2_CO_3_ (K_222_ = 10 μmol and K_2_CO_3_ = 6 μmol), a selective ^18^F-incorporation was observed affording [^18^F]FAMTO with a non-isolated RCY of 17% (entry 2, Table 2). As the “low base” approach gave promising results, we further reduced the amount of K_222_ and K_2_CO_3_ present in the reaction solution. Decreasing 2- and 4-times the amount of K_222_ and K_2_CO_3_, [^18^F]FAMTO was produced with a RCY between 16 and 23% (entries 3–4, Table 2).

Subsequent experiments were performed using the same amount of K_2_CO_3_ and K_222_ for [^18^F]fluoride ion elution and radiofluorination reaction allowing an easy transferability of manual procedures to automated synthesis modules. [^18^F]Fluoride ion was eluted from an anion exchange resin with a solution of K_2_CO_3_ (0.87 μmol) and K_222_ (1.5 μmol) dissolved in MeCN:H_2_O. Following the solvent evaporation, a solution of Cu(OTf)_2_(py)_4_ and **2** in 500 μL DMF was added to the dried residue of [^18^F]fluoride ion/K_222_/K_2_CO_3_ and the reaction mixture heated at 110 °C for 20 min. [^18^F]FAMTO was produced with non-isolated RCY of 32 ± 6% (entry 5). We next optimized the reaction with respect to solvent volume. Decreasing the volume of DMF from 500 to 300 and 200 μL (entries 6–7), the RCY of [^18^F]FAMTO remained the same. However a further decrease of the volume to 150 μL (entry 8), resulted in a non-isolated RCY drop from 32% to 11%. The synthesis, purification/reformulation and quality control time for the preparation of [^18^F]FAMTO are 75, 25 and 20 min, respectively. The radiosynthesis of [^18^F]FAMTO was achieved with an overall decay-corrected RCY of isolated [^18^F]FAMTO of 18 ± 2%, >99% RCP, >99% e.e. and 105 ± 39 GBq/μmol molar activity within 120 min of work, starting from 850 to 734 MBq of ^18^F-fluoride.

### 3.3 *In vitro* autoradiography

The *in vitro* binding of [^18^F]FAMTO was evaluated by autoradiography using pig adrenal, kidney and liver tissues. The incubation of adrenal gland sections with [^18^F]FAMTO showed a high specific binding with the signal of [^18^F]FAMTO completely blocked with 1 μM of MTO. Both liver and kidney organ samples were selected due to their known role in [^11^C]MTO metabolism and excretion [25]. Blocking studies revealed a high specific binding in the liver and kidney, organs known to be rich in cytochrome P450 enzymes.

### 3.4 *Ex vivo* biodistribution, metabolite analysis and *in vivo* PET imaging studies in rats

*Ex vivo* biodistribution data (Fig. 3A and Table S1), performed in healthy Sprague-Dawley rats, was used to assess the adrenal uptake as well as the adrenal-to-organ ratio. [^18^F]FAMTO showed high liver uptake in the first 10 min (%ID/g = 3.61 ± 1.53) followed by a slight decrease at 30 min (%ID/g = 2.16 ± 1.53) and 60 min (%ID/g = 1.68 ± 0.18). Adrenal uptake increased slowly during the first 30 min up to %ID/g = 6.2 ± 0.46, followed by a slow decrease up to %ID/g = 4.92 ± 0.70 at 60 min. Adrenal-to-liver ratio increased from 0.66 at 10 min to 2.8 at 30 min post-injection (Fig. 3A and Table S1).

Rats that received ETO (1 mg/kg) 10 min prior to injection of [^18^F]FAMTO showed a significant decrease in liver uptake (66%, *p* = 0.005, see Table S1) and a moderate decrease in the adrenal glands (39%, *p* = 0.03). Pre-treatment with ETO increased the ratio of %ID/g in adrenal to liver by 38% (*p* = 0.12) leading to an enhanced adrenal-to-liver PET signal compared to the non-pre-treatment group. An *in vivo* PET image of the adrenal uptake of [^18^F]FAMTO after pre-treatment with ETO (1 mg/kg) is shown in Figs. 3B and S4.

Plasma metabolite analysis revealed 13%, 8% and 2% of unchanged [^18^F]FAMTO at 10, 30 and 60 min post-injection, respectively. Two hydrophilic metabolites at retention times 3 and 21 min were observed (Fig. 4). The metabolite eluting at 3 min has been identified as the free acid [^18^F]**12** (Fig. S3). Urine metabolite analysis revealed only one metabolite in solution corresponding to [^18^F]**12** (Fig. S2).

### 3.5 Stability studies in human blood and plasma free fraction

Plasma-derived metabolites can cause a non-specific signal that reduces the specificity and quality of PET imaging endpoints. Metabolite studies of [11C]MTO or [18F]FETO in humans revealed that unchanged radiotracer accounted for 40% and 11% of total blood-borne radioactivity at 20 min post-injection, respectively and, considering the rapid in vivo metabolism of both radiotracers [30,37], we decided to perform [18F]FAMTO metabolite studies over a similar (20 min) timeframe for comparison. No degradation of [18F]FAMTO was observed after incubation in human blood for 20 min. The plasma free fraction of [18F]FAMTO was determined to be 37.55 ± 5.78% (mean ± SD, n = 5) by ultrafiltration.

## 4 Discussion

The development of the novel ^18^F radiotracer targeting adrenal gland enzymes represents a valuable advancement to the field of PA PET imaging. Our PET radiotracer development approach started from the structure-activity relationship analysis of MTO (K_i_ = 4.02 ± 1.87 nM), a potent inhibitor of CYP11B1 and CYP11B2 adrenal gland enzymes, and its derivatives bearing a halogen atom on the benzene ring (*e.g.* Br, I and F). [^11^C]MTO is a prototype radiotracer offering a rapid non-invasive procedure localizing aldosterone-producing adenomas [21,44]. MTO derivatives bearing an iodine, bromine or fluorine atom in the 4-position of the benzene ring (K_i_ = 8.7 ± 3.2 nM, 8.8 ± 2.4 nM and 8.15 ± 0.85 nM, respectively) have similar inhibitory activity to MTO, indicating that structural modification on the benzene ring does not significantly impact the affinity to the target's binding pocket [24,34,39]. These results encouraged us to attach a fluorine-18 atom at *para*-position of the MTO benzene ring generating a novel PET radiotracer ([^18^F]FAMTO, Fig. 1).

Initial investigations, focused on using previously recognised strategies to incorporate fluorine-18 into aromatic rings in a one-step reaction using synthetically accessible precursors such as aryl sulfonium salt **1** or an aryl boronic precursor **2** to obtain the *R*-enantiomer of [^18^F]FAMTO. The *R*-configuration of MTO (IC_50_ = 3.69 ± 1.92 nM) is a 130 times more potent an inhibitor than its *S*-enantiomer (IC_50_ = 492 ± 281 nM) [34].

Using the aryl sulfonium strategy, a benzylic 1H-imidazole derivative, ETO fragment, has been radiolabelled by Sander et al. in 31% RCY [41]. Applying the same conditions to **1**, the production of [^18^F]FAMTO was not achieved. Following a procedure developed by Mu et al. [45] that uses K_2_CO_3_ instead of KHCO_3_ and lowering the base concentration from 15 to 6 μmol, [^18^F]FAMTO was obtained in low non-isolated RCY (7 ± 2%).

An alternative radiolabelling strategy to produce [^18^F]FAMTO was subsequently investigated starting from the aryl boronic derivative **2**. When the dried residue of [^18^F]fluoride ion/K_222_/K_2_CO_3_ is taken up in a solution in DMF in the presence of copper-catalyst and **2**, the ^18^F-incorporation was not observed. A similar trend was observed by Neumaier et al. who demonstrated that large quantities of K_2_CO_3_ to elute [^18^F]fluoride ion from ion exchange cartridges impacts negatively on copper-mediated radiofluorination reactions, decreasing the RCY of the desired radiotracer [43]. Indeed using small aliquots of K_222_/K_2_CO_3_ in DMF obtained by dissolving the dried residue of [^18^F]fluoride ion/K_222_/K_2_CO_3_, a selective ^18^F-incorporation was observed affording [^18^F]FAMTO with an overall decay-corrected RCY of isolated [^18^F]FAMTO of 18%.

*In vitro* autoradiography on pig adrenal, liver and kidney sections, initially used to characterize the binding properties of [^18^F]FAMTO, demonstrated that [^18^F]FAMTO bound specifically to adrenal gland enzymes and that binding was completely blocked using 1 μM of MTO.

The simplicity of radiolabelling and encouraging results from *in vitro* studies justified further evaluation of the new tracer *in vivo*. Clinically desirable features for a PET radiotracer to be used in the identification of adenomas include high and specific radioactivity concentration in adrenal glands, rapid renal excretion, and low non-specific uptake of [^18^F]FAMTO in non-target tissues (*e.g.* liver, bone).

*In vivo* studies confirmed the high specificity of [^18^F]FAMTO uptake in adrenal glands showing a ratio of adrenal-liver uptake of 2.81 at 30 min post-injection, which is similar to the adrenal gland-to-liver ratio of [^11^C]MTO in rats (3.8 at 30 min) [32]. This ratio would be expected to be elevated in the presence of UAPA due to an overexpression of adrenal enzymes [8,46].

Image analysis of the rats administered with [^18^F]FAMTO was hampered by the high liver uptake masking the signal of the right adrenal gland. The pre-treatment of ETO 15 min prior to [^18^F]FAMTO injection lowered the liver uptake and increased the signal adrenal gland-to-liver of [^18^F]FAMTO (Figs. 3B and S4). Because of the known rapid metabolism of ETO *in vivo* [[47], [48], [49]], it is not expected that pre-treatment with 1 mg/kg ETO will cause complete blockade. However, *in vitro*, in the absence of MTO metabolism, 1 μM MTO was sufficient to fully block [^18^F]FAMTO specific binding.

High radioactivity was observed in the urine (urine-blood ratio was 4.36, 24.9 and 49.1 at 10, 30 and 60 min, respectively). Conversely, no uptake was visualised in bone, indicating an absence of [^18^F]FAMTO defluorination.

A metabolite study collecting rat blood samples after [^18^F]FAMTO injection revealed that 13% of radioactivity after 10 min was due to [^18^F]FAMTO (Fig. 4). The main metabolite in plasma and urine was identified to be the carboxylic acid of [^18^F]FAMTO ([^18^F]**12**, Figs. 4, S2 and S3). In analogy to the carboxylic acid derivative of MTO, we anticipate that compound **12** would also have negligible affinity for adrenal gland enzymes [34].Low metabolic stability of MTO and FETO has been observed *in vitro* and *in vivo* [30,37]. However, in humans [^18^F]FETO is metabolised faster than [^11^C]MTO. At 20 min post-injection, the unchanged [^18^F]FETO is 11% whereas [^11^C]MTO is 40% of total radioactivity [30,37]. The methyl-ester [^18^F]FAMTO might have the same profile as [^11^C]MTO, rather then the ethyl-ester [^18^F]FETO, however this hypothesis needs to be confirmed by determining [^18^F]FAMTO radiometabolic profile in humans. Blood-borne esterases might also produce radiometabolites to generate a non-specific background signal, reducing the specificity and quality of PET imaging endpoints. Because of this the stability of [^18^F]FAMTO was evaluated in human plasma revealing that [^18^F]FAMTO is highly stable in human plasma and blood.

## 5 Conclusion

[^18^F]FAMTO, a new ^18^F-labelled analogue of (*R*)-MTO was synthesised in a one-step radiofluorination procedure in good yields. The method utilises a synthetically accessible boronic ester precursor **2**, produced in a one-step reaction. A low base protocol is crucial for successful [^18^F]FAMTO labelling. *In vitro* and *in vivo* experiments have shown that [^18^F]FAMTO accumulates in adrenal glands, with good adrenal-to-liver ratios in rodents and a good radiometabolic profile with slow kinetics in the adrenals and rapid kinetics in the liver. The simplicity of radiolabelling and encouraging preclinical results justify progression to toxicology safety assessment studies and translation of [^18^F]FAMTO to healthy volunteers and patients with adrenal lesions of different adrenocortical and non-adrenocortical origin.

## Appendix A Supplementary data

Supplementary material Image 1
