# Supplementary material for: Development of [18F]FAMTO: A novel fluorine-18 labelled positron emission tomography (PET) radiotracer for imaging CYP11B1 and CYP11B2 enzymes in adrenal glands
Source: Nucl Med Biol. 2019 Jan-Feb;68-69:14–21. doi: 10.1016/j.nucmedbio.2018.11.002 (PMC6859501; doi:10.1016/j.nucmedbio.2018.11.002)
Supplement: Supplementary file 1 — Supplementary material [file mmc1.docx]

**Appendix A**

**SUPPLEMENTAL METHODS**

All reagents and consumables were purchased from Sigma Aldrich (Dorset, UK) or Fischer Scientific (Loughborough, UK), with the exception of (*S*)-[4-(phenylsulphanyl)phenyl]ethan-1-ol (AKos GmBH, Germany) and di-*tert*-butyl azodicarboxylate (DtBAD, ≥ 98.0%, Fluka). Male Sprague Dawley rats were purchased from Charles River, Margate, UK.

### Instrumentation

^1^H NMR and ^13^C proton decoupled NMR spectra were recorded at 400 MHz in 5 mm tubes on Bruker AV-400 spectrometers. Spectra were analysed with ACD/NMR processor (academic edition), V12.01. Chemical shifts (δ_H_ and δ_C_, respectively) are quoted in parts per million (ppm) and referenced to the appropriate residual solvent peak.^1^ Coupling constants (*J*) are reported to the nearest 0.1 Hz.

High resolution LC-ESI-MS was performed in positive ion mode on an Agilent 6520 Accurate-Mass Q-TOF LC/MS connected to an Agilent 1200 HPLC.

### Chromatography

Flash column chromatography was performed on silica gel (Merck Kieselgel 60 F254 230-400 mesh). Thin Layer Chromatography was performed on aluminium-backed plates pre-coated with silica (0.2 mm, 60 F254) which were developed using UV fluorescence or potassium permanganate staining. Radiochemical analysis and purification was performed manually on an Agilent 1200 LC equipped with a UV detector (λ=254 nm) and a β^+^-flow detector (NaI detector, B-FC-3200) coupled in series. Data were analyzed using Laura software (V.4.0.2.75 LabLogic Systems Ltd.).

Radioactive TLC (Silica Gel 60 F_254_ Coated Aluminum-Backed TLC strips, 10 cm length) were analysed on a Lab-Logic mini-Scan TLC reader with Laura software (V.4.0.2.75 LabLogic Systems Ltd.).

Chemical mass analysis was performed on an Agilent 1200 HPLC with an Agilent 6520 Accurate Mass QTOF LC/MS with ESI. Samples were injected directly. Spectra were recorded and analysed with MassHunter Workstation 2009 B.02.01 and Analysis 2009 B.03.01, respectively.

The synthesized FAMTO was used for comparison in the HPLC analysis of [^18^F]FAMTO and determine the calibration curve (supplemental Fig. 4).

Analytical HPLC method: chemical/radiochemical purity and serum stability of [^18^F]FAMTO were assessed using a Phenomenex Luna C18 column 5 µm (250 mm x 4.6 mm) with a flow rate of 2 mL/min, 20 µL loop and a gradient mobile phase: A = H_2_O, B = MeCN: 0-3 min =70% of A, 3-14 min = 70% to 20% of A, 14-16 min= 20% of A, 16-18 min= 20% to 70% of A, 18-20 min= 70% of A. t_R_ (**4**) = 11:50 min.

Semipreparative HPLC methods: metabolite analysis and purification of [^18^F]FAMTO were assessed using a Phenomenex Luna C18 column 10 µm (250 x 4.6 mm) with a flow rate of 5 mL/min and 1 mL loop. Gradient mobile phase: A = H_2_O, B = MeCN, gradient: 0-5 minutes = 70% of A, 5-26 minutes = 70% to 20% A, 26-30 minutes = 20% of A, 30-34 min =20% to 70% of A, 34-40 min = 70% of A. t_R_ (**4**) =21:40 min.

Solid phase extraction (SPE) cartridge specifications: Sep-Pak Accell Plus QMA SPE Cartridge, 130 mg, 37-55 μm (cat. no. WAT023525, Waters).

### General synthesis of compounds 3-6

To a stirred solution of **11** (1.1 mmol, 1 equiv) and triphenylphosphine (1.2 equiv) in 3 mL of anhydrous THF was added a solution of enantiopure derivatives (*S*)-**7-10** (1 equiv) in 2 mL of anhydrous THF under argon atmosphere at room temperature. Then a solution of di-*tert*-butyl azodicarboxylate (1.2 equiv) in 2 mL of anhydrous THF was added to the mixture dropwise at 0 °C in 10 minutes. The mixture was allowed to warm up until room temperature within two hours and was stirred overnight. Solvent was evaporated under reduced pressure and 5 mL of ether were added to the mixture that was let stirred for two hours. The mixture was filtered and the solid was washed three times with 1 mL of ether. The filtered solution was concentrated under reduced pressure and purified by flash chromatography (Hexane:Ether:Diisopropylamine = 40:30:1).

**3** was obtained as a yellow oil (114 mg, 45%). Rf = 0.45 (ether:diisopropylamine = 10:0.1); ^1^H NMR (CDCl_3_, 400 MHz) δ 7.70 (s, 1H), 7.66 (s, 1H), 7.31 – 7.18 (m, 3H), 7.15 – 7.08 (m, 2H), 6.29 (q, *J* = 7.1 Hz, 1H), 3.74 (s, 3H), 1.80 (d, *J* = 7.1 Hz, 3H). ^13^C NMR (CDCl_3_, 100.6 MHz) δ 159.68 (*C*O), 133.77 (*C*arH), 130.70 (*C*arH), 130.18 (*C*arH), 128.11 (C*C*arHN), 123.15 (*C*arH), 122.45 (*C*arH), 54.26 (*C*HCH_3_), 50.42 (O*C*H3), 20.87 (C*C*H3). MS (ESI+): m/z [C_13_H_14_N_2_O_2_ + H]^+^ calculated = 231.1133, experimental= 231.1151.

**4** was obtained as yellow oil, (110 mg, 40%). Rf = 0.39 (ether:diisopropylamine = 10:0.1), Rf= 0.70 (dichloromethane:methanol:TFA= 8:2:0.05).; ^1^H NMR (CDCl_3_, 400 MHz) δ 7.70 (s, 1H), 7.66 (s, 1H), 7.11-7.08 (m, 2H), 6.97-6.93 (m, 2H), 6.26 (q, *J* = 7.1, 1H) 3.74 (s, 3H), 1.78 (d, *J* =7.1, 3H). ^13^C NMR (CDCl_3_, 100.6 MHz) δ 162.48 (*C*F), 159.64 (*C*O), 138.59 (*C*arH), 137.33 (*C*arH), 135.94 (*C*arH), 127.02 (C*C*arHN), 126.93(*C*arH), 121.23(*C*arH), 114.85 (*C*arH), 114.63(*C*arH), 53.70 (*C*HCH_3_), 50.47 (O*C*H3), 21.28 (C*C*H3). MS (ESI+): m/z [C_13_H_13_FN_2_O_2_ + H]^+^ calculated = 249.1034, experimental= 249.1035.

**5** was obtained as a brown oil (227 mg, 61%). Rf = 0.29 (ether:diisopropylamine = 10:0.1); ^1^H NMR (CDCl_3_, 400 MHz) δ 7.68 (m, 2H), 7.30 – 7.16 (m, 7H), 7.03 – 7.01 (m, 2H), 6.26 (q, *J* = 7.1 Hz, 1H), 3.73 (s, 3H), 1.79 (d, *J* = 7.1 Hz, 3H). ^13^C NMR (CDCl_3_, 100.6 MHz) 159.65 (*C*O), 138.69 (*C*arH), 138.64 (*C*arH), 137.24 (*C*arH), 135.38 (*C*arH), 133.57 (*C*arH), 130.87 (*C*arH), 129.42 (*C*arH), 128.31 (*C*arH), 126.57 (C*C*arHN), 126.01 (*C*arH), 121.31 (*C*arH), 53.88 (*C*HCH_3_), 50.48 (O*C*H3), 21.10 (C*C*H3). MS (ESI+): m/z [C_19_H_18_N_2_O_2_S + H]^+^ calculated= 339.1162, experimental= 339.1160.

**6** was obtained as yellowish oil (126 mg, 37%). Rf = 0.39 (ether:diisopropylamine = 10:0.1). ^1^H NMR (CDCl_3_, 400 MHz) δ 8.81 (s, 1H), 7.82 (s, 1H), 7.46 – 7.42 (m, 2H), 7.13 –7.09 (m, 2H), 6.35 (q, *J* = 7.1 Hz, 1H), 3.81 (s, 3H), 1.90 (d, *J* = 7.1 Hz, 3H). ^13^C NMR (CDCl_3_, 100.6 MHz) δ 158.13 (*C*O), 138.31 (*C*arH), 132.44 (*C*arH), 131.20 (*C*arH), 128.27 (*C*arH), 127.17 (C*C*arHN), 57.03 (*C*HCH_3_), 52.55 (O*C*H3), 22.04 (C*C*H3). MS (ESI+): m/z [C_13_H_13_^79^BrN_2_O_2_ + H]^+^ calculated= 309.0233, experimental= 309.0228, m/z [C_13_H_13_^81^BrN_2_O_2_ + H]^+^ calculated= 311.0214, experimental= 311.0205.

### Synthesis of (R)-(4-(1-(5-(methoxycarbonyl)-1H-imidazol-1-yl)ethyl)phenyl)diphenylsulfonium trifluoromethanesulfonate (1)

Compound **1** was synthesized following the procedure reported by Sander *et al.* using **5** (88 mg, 0.26 mmol, 1 equiv), TFSA (23 μL, 0.26 mmol, 1 equiv), diaryliodonium trifluoromethanesulfonate (112 mg, 0.26 mmol, 1 equiv) and copper benzoate (4 mg, 13 µmol, 0.05 equiv) in 1 mL chlorobenzene.^2^ **1** was obtained as a brown oil (93 mg, 53%). Rf = 0.40 (dichloromethane:methanol = 9:1). ^1^H NMR (CDCl_3_, 400 MHz,) δ 7.85-7.83 (m, 2H), 7.72 – 7.60 (m, 12H), 7.39 – 7.37 (m, 2H), 6.34 (q, *J* = 7.1 Hz, 1H), 3.69 (s, 3H), 1.86 (d, *J* = 7.1 Hz, 3H). ^13^C NMR (CDCl_3_, 100.6 MHz) δ 159.66 (*C*O), 140.07 (*C*arH), 138.82 (*C*arH), 137.21 (*C*arH), 127.83 (C*C*arHN), 126.97 (*C*arH), 125.23 (*C*arH), 121.30 (*C*F_3_), 53.88 (*C*HCH_3_), 50.48 (O*C*H_3_), 21.10 (C*C*H_3_). MS (ESI+): m/z [C_25_H_23_N_2_O_2_S]^+^ calculated= 415.1475, experimental= 415.1472.

### Synthesis of methyl (R)-1-(1-(4-(4,4,5,5-tetramethyl-1,3,2-dioxaborolan-2-yl)phenyl)ethyl)-1H-imidazole-5-carboxylate (2)

In a glovebox under argon atmosphere **6** (126 mg, 0.41 mmol, 1 equiv), bis(pinacolato)diboron (114.5 mg, 0.45 mmol, 1.1 equiv), potassium acetate (120.7 mg, 1.23 mmol, 3 equiv) and [1,1'-bis(diphenylphosphino)ferrocene]palladium(II) dichloride (43.9 mg, 0.06 mmol) were placed in a 20 mL vial equipped with a stir bar. Anhydrous DMSO (2 mL) is added to the mixture and the vial was sealed with a Teflon cap and taken out of the glovebox. The vial was left stirring at 80 °C for 15h. Then the reaction mixture was cooled to room temperature, diluted with 3.7 mL of ether and filtered through celite. The organic layer was washed with H_2_O (3x5.6mL). The combined organic fractions were dried over anhydrous MgSO_4_ and concentrated in vacuum. The product was washed with a saturated solution of NaHCO_3_ (2x4 mL), and **2** obtained as brownish oil (120 mg, 82%, n=3). Rf = 0.4 (ether:diisopropylamine = 10:0.1). ^1^H NMR (CDCl_3_, 400 MHz) δ 8.03 (s, 1H), 7.74-7.72 (m, 1H), 7.42-7.40 (m, 2H), 7.14-7.12 (m, 2H), 6.33 (q, *J* = 7 Hz, 1H), 3.77 (s, 3H), 1.83 (d, *J* = 7 Hz, 3H), 1.27 (s, 12H). ^13^C NMR (CDCl_3_, 100.6 MHz) δ 170.84 (*C*O), 135.58 (*C*arH), 132.19 (*C*arH), 128.00 (C*C*arHN), 125.65 (*C*arH), 84.02 (*C*CH_3_), 56.83 (*C*HCH_3_), 52.09 (O*C*H_3_), 24.85 (C*C*H_3_), 21.64 (C*C*H_3_). MS (ESI+): m/z [C_19_H_25_BN_2_O_4_ + H]^+^ calculated= 357.1985, experimental= 357.1976.

### Synthesis of (R)-1-(1-(4-fluorophenyl)ethyl)-1H-imidazole-5-carboxylic acid (12)

A solution of **4** (40 mg, 0.161 mmol) was stirred in a mixture of 1 mL methanol and 1 mL of a solution 2N NaOH overnight at room temperature. The reaction mixture was then acidified by addiction of 1 M HCl until pH≈7 and extracted with ethyl acetate. **12** was achieved as yellowish solid (15.1 mg, 40%). Rf= 0.45 (dichloromethane:methanol:TFA= 8:2:0.05). ^1^H NMR (DMSO-d_6_, 400 MHz) δ 9.43 (bs, 1H), 8.27 (s, 2H), 7.36-7.34 (m, 2H), 7.23-7.19 (m, 2H), 3.44 (q, *J* = 8 Hz, 1H), 1.89 (d, *J* = 8 Hz, 3H). ^13^C NMR (DMSO-d_6_, 150 MHz) δ 161.39 (*C*F), 160.95 (*C*O), 159.89 (*C*arH), 138.77 (*C*arH), 137.16 (*C*arH), 129.06 (*C*arH), 128.97 (C*C*arHN), 124.30 (*C*arH), 116.15 (*C*arH), 115.93 (*C*arH), 56.43 (*C*HCH_3_), 19.03(C*C*H_3_). MS (ESI+): m/z [C_12_H_11_FN_2_O_2_ + H]^+^ calculated= 235.0877, experimental= 235.0873.

### Enantiomeric purity

Optical rotations were measured at 25 ° C on an Applied Photophysics Ltd Chirascan Plus spectrometer (Pharmaceutical Optical & Chiroptical Spectroscopy Facility, Guy’s Campus, KCL, London) in a 0.5 mm strain-free rectangular cells in the 400-190 nm region. The following parameters were employed: 1 nm spectral bandwidth, 1 nm step-size and 1.5 s measurement time-per-point.

Enantiomeric purity of (*R*)-**ETO** (Sigma), (*R*)-**2**, (*R*)-**4**, (*R*)-**5** and (*R*)-[^18^F]**FAMTO** was performed in a chiral Lux 5 µm Cellulose-3 column (250 x 4.6 mm) at a flow of 1 mL/min (20 µL loop). Mixture of analytical grade methanol and a solution 20 mM ammonium bicarbonate with 0.1% of diethylamine (80:20) was used as mobile phase through isocratic elution. The UV lamp was set at 220 nm.

### Anaesthesia

Dynamic PET imaging of all rats was performed under isoflurane anaesthesia. The oxygen was kept at 1 L/min flow rate with isoflurane levels of 5% for induction and termination, while for maintenance the isoflurane rate was set to 1.5-3% with constant breathing and temperature monitoring.

### In vivo biodistribution

The saline-dissolved reformulated radiotracer was injected under isoflurane anaesthesia in 0.2–3 MBq, corresponding to an administered dose of 0.5–16 ng/kg, and >100 GBq/μmol molar radioactivity via tail vein cannulation, followed by a 0.15 mL saline flush. Immediately after imaging, the animals were culled. Radioactivity counting was performed with a gamma counter (LKB Wallac, PerkinElmer, UK) for in vivo biodistribution.

### PAINS filter

FAMTO passed through a pan assay interference compounds (PAINS) filter (http://zinc15.docking.org/patterns/home/).^3^

**REFERENCES**

1. Fulmer, G. R.; Miller, A. J. M.; Sherden, N. H.; Gottlieb, H. E.; Nudelman, A.; Stoltz, B. M.; Bercaw, J. E.; Goldberg, K. I. NMR Chemical Shifts of Trace Impurities: Common Laboratory Solvents, Organics, and Gases in Deuterated Solvents Relevant to the Organometallic Chemist. *Organometallics* **2010**, *29*, 2176-2179.

2. Sander, K.; Gendron, T.; Yiannaki, E.; Cybulska, K.; Kalber, T. L.; Lythgoe, M. F.; Arstad, E. Sulfonium Salts as Leaving Groups for Aromatic Labelling of Drug-like Small Molecules with Fluorine-18. *Scientific Reports* **2015**, *5*.

3. Aldrich, C.; Bertozzi, C.; Georg, G. I.; Kiessling, L.; Lindsley, C.; Liotta, D.; Merz, K. M., Jr.; Schepartz, A.; Wang, S. The Ecstasy and Agony of Assay Interference Compounds. *J. Med. Chem.* **2017**, *60*, 2165-2168.


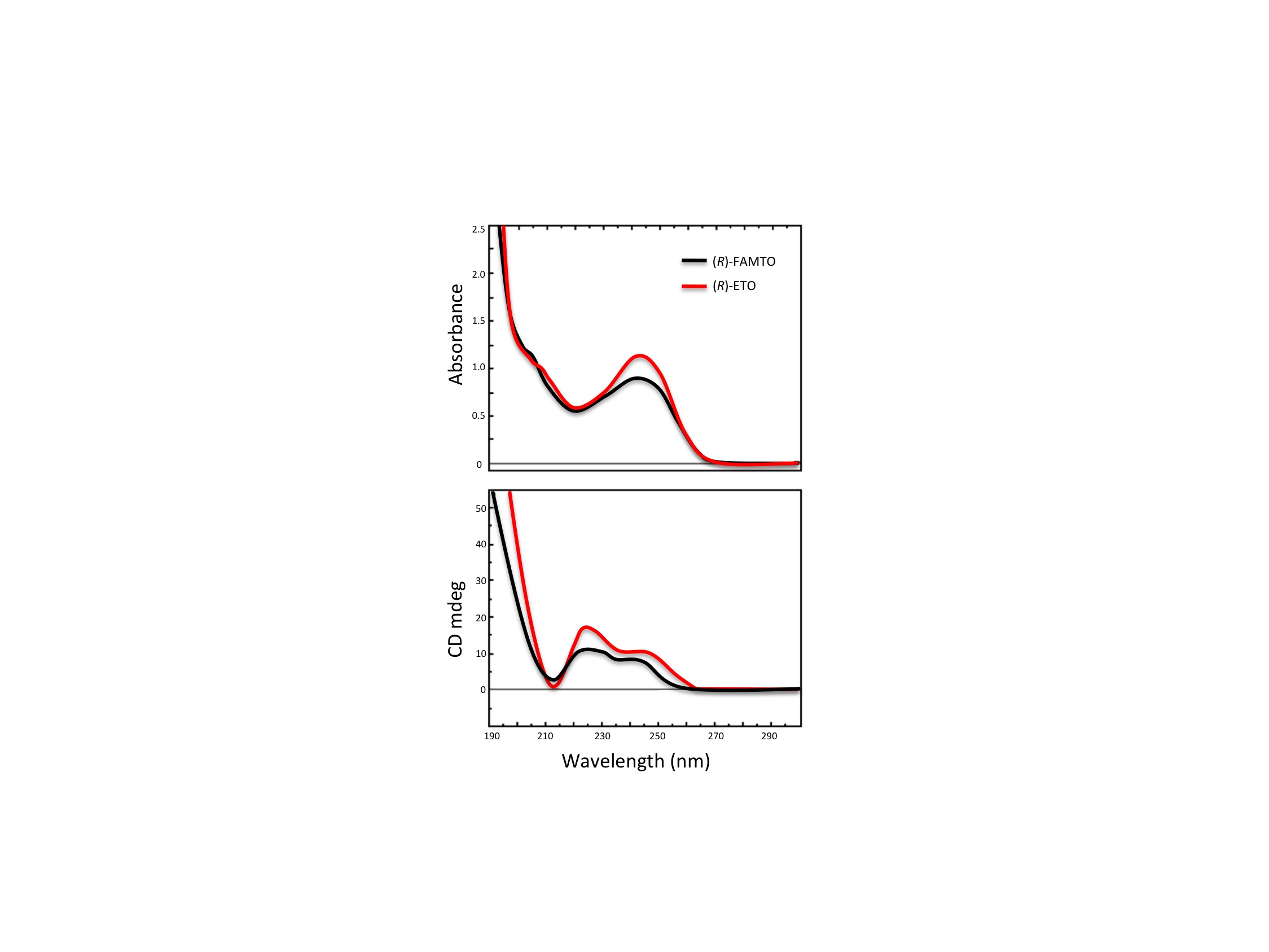


**Figure S1.** Measured UV and CD spectra in MeCN of (*R*)-FAMTO (0.33 mg/ml) and (*R*)-ETO (0.5 mg/mL).


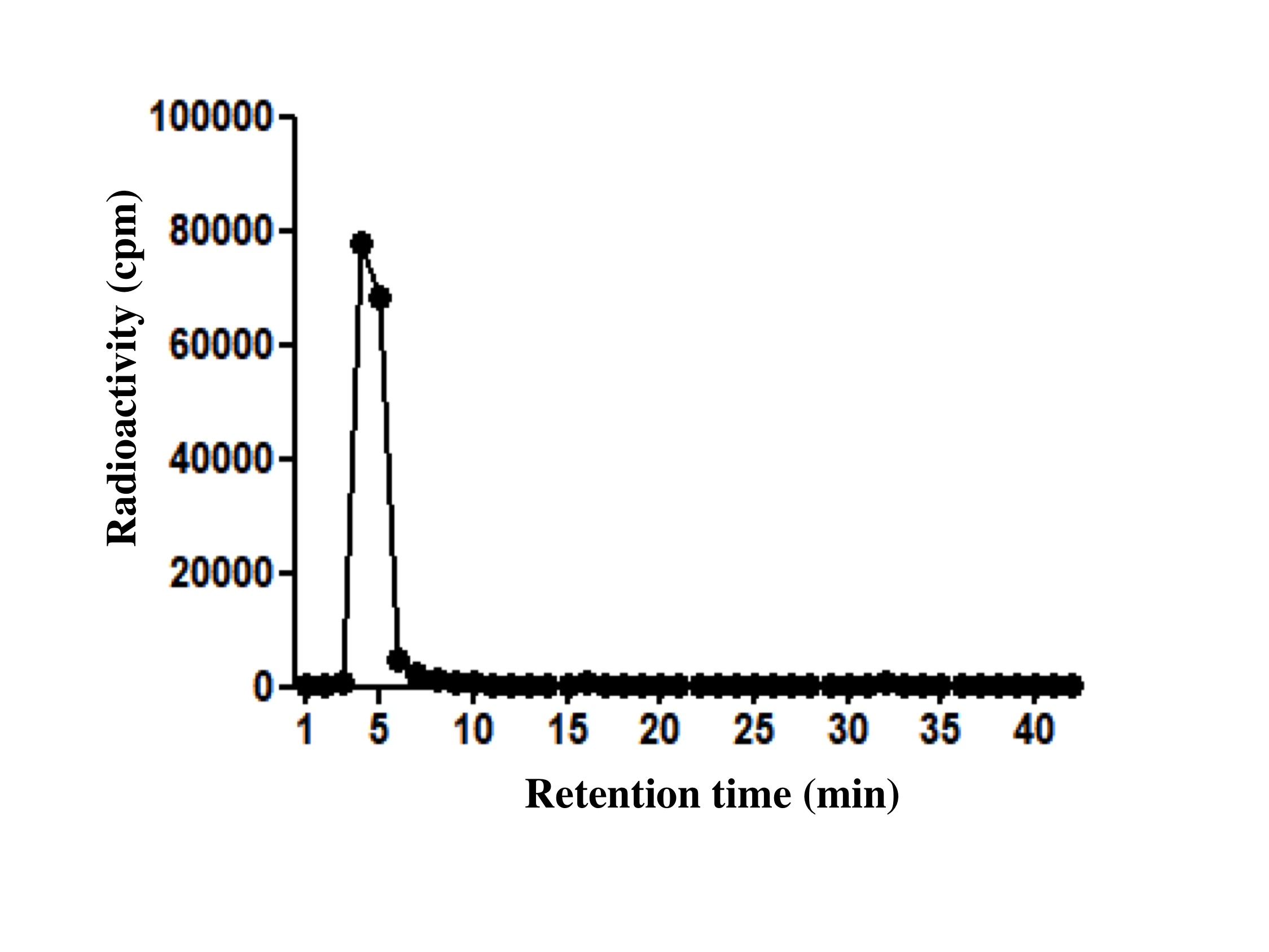


**Figure S2.** Metabolite analysis in urine after 30 minutes [^18^F]FAMTO post-injection.

**Figure S3.** Metabolism of [^18^F]FAMTO *in vivo*.

**FIGURE S4.** In vivo PET imaging – transaxial view - of a male Sprague Dawley rats showing adrenal uptake of [^18^F]FAMTO after ETO (1 mg/kg) pre-treatment.

**TABLE S1** Biodistribution of [^18^F]FAMTO in male Sprague Dawley rats at 15, 30, 60 minutes and rats pretreated with ETO (1 mg/Kg) 15 min before the injection of [^18^F]FAMTO.

|  | [^18^F]FAMTO | | | | | | | | | Pre-treatment with ETO 15 minutes before the injection of [^18^F]FAMTO | | | |
| --- | --- | --- | --- | --- | --- | --- | --- | --- | --- | --- | --- | --- | --- |
| **Organ** | **15 min** | | | **30 min** | | | **60 min** | | | **30 min** | | | |
|  |  |  |  |  |  |  |  |  |  |  | |  |  |
| **Blood** | 0.61 | ± | 0.19 | 0.25 | ± | 0.19 | 0.22 | ± | 0.05 | | 0.44 | ± | 0.11 |
| **Heart** | 0.29 | ± | 0.06 | 0.17 | ± | 0.06 | 0.15 | ± | 0.03 | | 0.21 | ± | 0.04 |
| **Lung** | 0.39 | ± | 0.07 | 0.23 | ± | 0.07 | 0.19 | ± | 0.02 | | 0.25 | ± | 0.06 |
| **Liver** | 3.61 | ± | 1.53 | 2.16 | ± | 1.53 | 1.68 | ± | 0.18 | | 0.96 | ± | 0.15 |
| **Intestine** | 0.30 | ± | 0.08 | 0.44 | ± | 0.08 | 0.62 | ± | 0.16 | | 0.25 | ± | 0.02 |
| **Adrenal** | 2.41 | ± | 0.46 | 6.21 | ± | 0.46* | 4.92 | ± | 0.70 | | 3.84 | ± | 0.61* |
| **Kidney** | 1.60 | ± | 0.34 | 0.95 | ± | 0.34 | 0.52 | ± | 0.14 | | 0.32 | ± | 0.00 |
| **Spleen** | 0.15 | ± | 0.02 | 0.10 | ± | 0.02 | 0.09 | ± | 0.01 | | 0.11 | ± | 0.02 |
| **Testis** | 0.11 | ± | 0.03 | 0.10 | ± | 0.03 | 0.08 | ± | 0.02 | | 0.17 | ± | 0.03 |
| **Bone** | 0.06 | ± | 0.09 | 0.06 | ± | 0.09 | 0.09 | ± | 0.05 | | 0.09 | ± | 0.02 |
| **Brain** | 0.06 | ± | 0.02 | 0.08 | ± | 0.02 | 0.05 | ± | 0.00 | | 0.08 | ± | 0.01 |
| **Cerebellum** | 0.09 | ± | 0.02 | 0.08 | ± | 0.02 | 0.06 | ± | 0.01 | | 0.10 | ± | 0.01 |

%ID/g values (mean ± SE) with n = 3 rats per group. *p* value was determined by using two-tailed paired Student’s t test for the adrenal gland uptake in control rats (30 min) versus rats pre-treated with ETO. ^*^*p* < 0.05.

**TABLE S2** Retention time and enantiomeric excess (e.e.).

|  | t_R_ | e.e. |
| --- | --- | --- |
| (*R*)-ETO | 5:25 | >99% |
| (*R*)-MTO | 5:22^a^ | n.d. |
| (*R*)-2 | 11:20 | >99% |
| (*R*)-4 | 5:08 | >95% |
| (*R*)-5 | 8:05 | >99% |
| (*R*)-[^18^F]FAMTO | 5:15 | >99% |

^*^From Phenomenex (2017): *http://www.phenomenex.com/Application/Detail/20304?alias=etomidate&returnURL=/Application/Search* (Accessed 11 Dec 2017).
